# Cortical and subcortical morphometric changes and their relation to cognitive impairment in isolated REM sleep behavior disorder

**DOI:** 10.1007/s10072-023-07040-z

**Published:** 2023-09-05

**Authors:** Christiane Mala, Filip Havlík, Josef Mana, Jiří Nepožitek, Simona Dostálová, Evžen Růžička, Karel Šonka, Jiří Keller, Robert Jech, Petr Dušek, Ondrej Bezdicek, Radim Krupička

**Affiliations:** 1 Department of Biomedical Informatics, Faculty of Biomedical Engineering, Czech Technical University in Prague, Prague, Czech Republic; 2 Department of Neurology and Centre of Clinical Neuroscience, First Faculty of Medicine, Charles University and General University Hospital, Prague, Czech Republic; 3 Department of Radiology, Na Homolce Hospital, Prague, Czech Republic

**Keywords:** Mild cognitive impairment, Prodromal, Synucleinopathy, Cognition, Trail Making Test, REM sleep without atonia

## Abstract

**Objective:**

To date, very few studies have focused on structural changes and their association with cognitive performance in isolated REM sleep behaviour disorder (iRBD). Moreover, the results of these studies are inconclusive. This study aims to evaluate differences in the associations between brain morphology and cognitive tests in iRBD and healthy controls.

**Methods:**

Sixty-three patients with iRBD and thirty-six controls underwent MRI with a 3 T scanner. The cognitive performance was assessed by a comprehensive neuropsychological battery. Based on performance, the iRBD group was divided into two subgroups with (iRBD-MCI) and without mild cognitive impairment (iRBD-NC). The high-resolution T1-weighted images were analysed using an automated atlas segmentation tool, voxel-based (VBM) and deformation-based (DBM) morphometry to identify between-group differences and correlations with cognitive performance.

**Results:**

VBM, DBM and the comparison of ROI volumes yielded no significant differences between iRBD and controls. In the iRBD group, significant correlations in VBM were found between several cortical and subcortical structures primarily located in the temporal, parietal, occipital lobe, cerebellum, and basal ganglia and three cognitive tests assessing psychomotor speed and one memory test. Between-group analysis of cognition revealed a significant difference between iRBD-MCI and iRBD-NC in tests including a processing speed component.

**Conclusions:**

iRBD shows deficits in several cognitive tests that correlate with morphological changes, the most prominent of which is in psychomotor speed and visual attention as measured by the TMT-A and associated with the volume of striatum, insula, cerebellum, temporal lobe, pallidum and amygdala.

**Supplementary Information:**

The online version contains supplementary material available at 10.1007/s10072-023-07040-z.

## Introduction

As a result of neuropathological changes, patients with idiopathic rapid eye movement sleep behavior disorder (iRBD) suffer from neuropsychiatric symptoms such as anxiety, depression, or apathy and cognitive impairment [1]. Executive functions with memory appear to be the most impaired cognitive domains [2], but deficits in other domains are also reported [1]. These deficits are so prevalent that a higher proportion of iRBD patients are diagnosed with mild cognitive impairment (MCI) than de novo patients with Parkinson's disease (PD) without RBD [3].

Recently, more attention has been focused on the combination of imaging methods and cognitive tests to elucidate the neuroanatomical basis of cognitive impairment in iRBD [4, 5], however, studies combining structural imaging techniques with cognitive functions are few and yield inconsistent results. Specifically, they vary considerably with respect to the region of atrophy and associated cognitive deficit. For example, Rahayel et al. [6] revealed an association between attention/executive functions and thinning in several regions of frontal (medial superior, dorsolateral paracentral, sensorimotor), temporal (fusiform, lingual), and occipital (cuneus) cortices, and associations were also found for visuospatial abilities (frontal, temporal, insular, parietal, occipital) and learning/memory (temporal, insular, occipital). Pereira et al. [4] found significant correlations only for memory and thinning in the left superior temporal, left caudal middle frontal, right superior frontal, right lateral occipital gyri, and for visuospatial functions and thinning in the left fusiform, right supramarginal gyri. And in contrast to the above studies, Campabadal et al. [7] failed to detect significant associations between brain atrophy and cognitive performance. There are also other studies [8] that have sought to find an association between brain-morphometric changes and cognition, however, the interpretation of the results is limited due to the use of cognitive screening tests only.

The inconclusive results may be partially explained by limited sample sizes, which usually include less than 30 patients. In addition, reliance on a single structural imaging technique is common, as is the use of only some neuropsychological subscores and the omission of cognitive status, i.e., MCI. In this situation, further exploratory research involving larger samples, broader range of psychological variables, and more structural imaging techniques is needed to obtain more reliable information about the neuroanatomical basis underlying cognitive impairment in iRBD.

Therefore, the present study aims to address these shortcomings by (1) comparing morphometric changes between iRBD patients and healthy controls (HC), (2) comparing the level of cognitive functioning between iRBD and HC, and (3) examining the association between morphometric changes and cognitive performance, all based on a comprehensive neuropsychological battery and several structural imaging techniques performed on a relatively large sample of patients. As REM without atonia (RWA) has been hypothesized to be an indicator of disease progression and may indicate the severity of cognitive deficit [9], a secondary aim of the study is also to test the association of RWA with performance in cognitive tests.

## Methods

### Participants

iRBD patients were diagnosed at the Department of Neurology, First Faculty of Medicine, Charles University and General University Hospital in Prague using video-polysomnography (video-PSG) according to the International classification of sleep disorders, third edition [10]. The inclusion criteria were age over 50 years and over 8 years of education. The exclusion criteria were an overt neurodegenerative disease, dementia, narcolepsy, epilepsy, encephalitis, drug-induced RBD, head injury, or focal brain lesion indicative of secondary RBD. Consistent with previous studies [11], patients were classified as iRBD with mild cognitive impairment (iRBD-MCI) if their *z*-scores adjusted for age, sex, and education were below -1.5 in at least two tests in one domain, or in at least one test per domain in at least two domains, and as iRBD with normal cognition (iRBD-NC) in other cases. The HC group was chosen as the reference group because of comparability and lack of local normative data for some of the tests.

HCs were recruited from the general community via social media and web advertisement and were matched to have age, education, and sex comparable to the iRBD group. All controls underwent a detailed medical interview, neuropsychological examination and video-PSG. Exclusion criteria were neurological or psychiatric disease (e.g., Parkinson’s disease, epilepsy, schizophrenia, depression, anxiety, history of stroke or major head trauma), alcohol or drug addiction, chemotherapy or radiotherapy, major somatic illness (cancer, symptomatic coronary heart disease etc.), sleep disorders (untreated sleep apnea, insomnia, narcolepsy), major hearing and vision problems, and cognitive deficit.

All patients and HCs provided written informed consent. The study was approved by the Ethics Committee of the General University Hospital in Prague under the number 11/15.

### Image acquisition

The examination was performed on a 3 T MRI scanner (Siemens Skyra 3 T, Siemens Healthcare, Erlangen, Germany) with a 32-channel head coil.

Morphometry analysis was performed on T1-weighted 3D Magnetization-Prepared Rapid Acquisition with Gradient Echo (MPRAGE) images in the axial plane with the following acquisition parameters: repetition time (TR), 2,200 ms; echo time (TE), 2.4 ms; inversion time (TI) 900 ms; flip angle (FA) 8°; field of view (FOV) 230 × 197 × 176 mm; spatial resolution 1 mm isotropic.

### Image preprocessing

The preprocessing and segmentation of T1 weighted images were done with the Computational Anatomy Toolbox software, version 12.7 [CAT; 12] implemented in statistical parametric mapping software (SPM, version 7771) [13] in Matlab [14].

The segmentation was done by Hammers atlas [15]. The outcome of the segmentation process was the raw data of total intracranial volume (TIV), total grey matter (GM) and total white matter (WM) for each subject as well as the absolute values for 34 different brain regions in the Hammers Atlas, each region divided into left and right hemisphere values. Regional volumes of interest were adjusted to TIV by dividing the raw volume of each region by the TIV of the patient. A visual quality check was done by reviewing one slice of every brain to find obvious artefacts in the scans and incorrectly oriented images. Data homogeneity was checked by the CAT data quality batch. The quality of segmentation was checked for every T1-weighted image and a minimum of C + in all quality parameters was accepted.

For voxel-wise analysis, the modulated, normalized grey matter segments were smoothed using a Gaussian kernel with an 8 mm^3^ full width at half maximum.

Voxel-based morphometry (VBM) was performed with the smoothed grey matter volume maps. Deformation-based morphometry (DBM) was performed with the smoothed Jacobian determinants. Smoothing was performed with the same settings as for VBM.

### Video-polysomnography

Video-PSG was performed using a digital PSG system (RemLogic, version 3.4.1, Embla Systems) and consisted of standard montage according to the American Academy of Sleep Medicine (AASM) recommendation [16] supplemented by bilateral flexor digitorum superficialis (FDS) muscle electromyography (EMG). All features on video-PSG were analyzed visually. The quantification of RWA was based on the SINBAR recommendations [17]. The calculated parameters representing severity of RWA included SINBAR score (the percentage of REM sleep referring to the amount of any chin and/or bilateral FDS phasic REM sleep-related EMG activity), tonic RWA index (percentage of REM sleep with sustained increase in chin EMG activity relative to total REM sleep duration), phasic RWA index (percentage of REM sleep with bursts of chin EMG activity relative to total REM sleep duration), and mixed RWA index (percentage of REM sleep with chin EMG bursts superimposed on sustained EMG activity) [18].

### Neuropsychological and clinical assessment

To evaluate motor and neuropsychiatric symptoms, all iRBD patients and HC were clinically examined using Movement Disorders Society-Unified Parkinson’s Disease Rating Scale, part III (MDS-UPDRS-III) [19], and completed the following questionnaires: Beck Depression Inventory, Second Edition (BDI-II) [20], State-Trait Anxiety Inventory (STAI) [21], and Epworth Sleepiness Scale, part II (ESS) [22].

The Montreal Cognitive Assessment (MoCA) was used to screen cognition and the Czech adaptation of the National Adult Reading Test (CART/NART) [23] to estimate premorbid intelligence level. Furthermore, both groups were evaluated by a complex neuropsychological battery covering six cognitive domains (attention/working memory, executive functions, language, episodic memory, visuospatial functions and processing speed/psychomotor speed), see Supplemental Table 1 and Wenke et al. [24] for a detailed description.

### Statistical analysis

Differences between groups in socio-demographic and clinical characteristics were tested by parametric (one-way ANOVA, two-sample t-test) or by nonparametric (Kruskal–Wallis, Mann–Whitney, chi-square) tests as appropriate.

Statistical analysis of VBM, DBM and ROI volumes were performed in SPM12/CAT12. The between-group comparison was performed by using the general linear model with age and sex as covariates. For VBM the total intracranial volume (TIV) was used as third covariate. The statistical map for between-group comparison was thresholded at p < 0.05 statistical level corrected by family-wise error (FWE). The correlations between cognitive tests and brain morphology for all VBM, DBM and ROI analysis were performed in CAT12 using as an additional covariate, to the above described, the results of the cognitive test. Thresholds and corrections of the statistical maps were the same as for between group comparison.

Raw neuropsychological scores that were not normally distributed according to the Shapiro–Wilk test were first transformed to follow a normal distribution. One neuropsychological variable was missing in three HCs and two in one iRBD. To avoid sample reduction, these values were imputed with the Hmisc R package [25]. Multiple regression analyses with predictors of age, sex, and education were performed to calculate z-scores for each participant. Only the HC group was used to estimate the regression parameters and the ensuing z-scores, thus these represent the relative deficit of each participant with respect to age, sex and education-matched healthy control. These *z*-scores were used to compare all groups by nested ANOVA and for correlations with RWA. As analysis of raw scores can be challenging to interpret and averaging raw scores may lead to failure to detect meaningful differences [26], principal component analysis (PCA) with varimax rotation was performed on the raw scores of the total sample. Subsequently, component scores for each subject were computed and nested ANOVA was performed again on these scores to determine the differences in cognitive domains identified by the PCA. In all ANOVAs, the group was used as a nesting fixed factor, MCI status as a nested fixed factor, and *z*-score or PCA component scores as response variables. Benjamini–Hochberg correction was used in all psychological analyses to address the multiple comparisons. Tukey HSD post hoc test was performed with significant results.

In morphometric analyses, groups were treated as with (iRBD-NC vs iRBD-MCI vs HC) or without inclusion of MCI status (iRBD vs HC), whereas in psychological analyses, all groups (iRBD-NC vs iRBD-MCI vs HC vs iRBD vs HC) were compared simultaneously.

Statistical analyses were performed using RStudio [27] and Matlab Toolbox CAT [12].

## Results

### Sociodemographic and clinical data

**Fig. 1:**
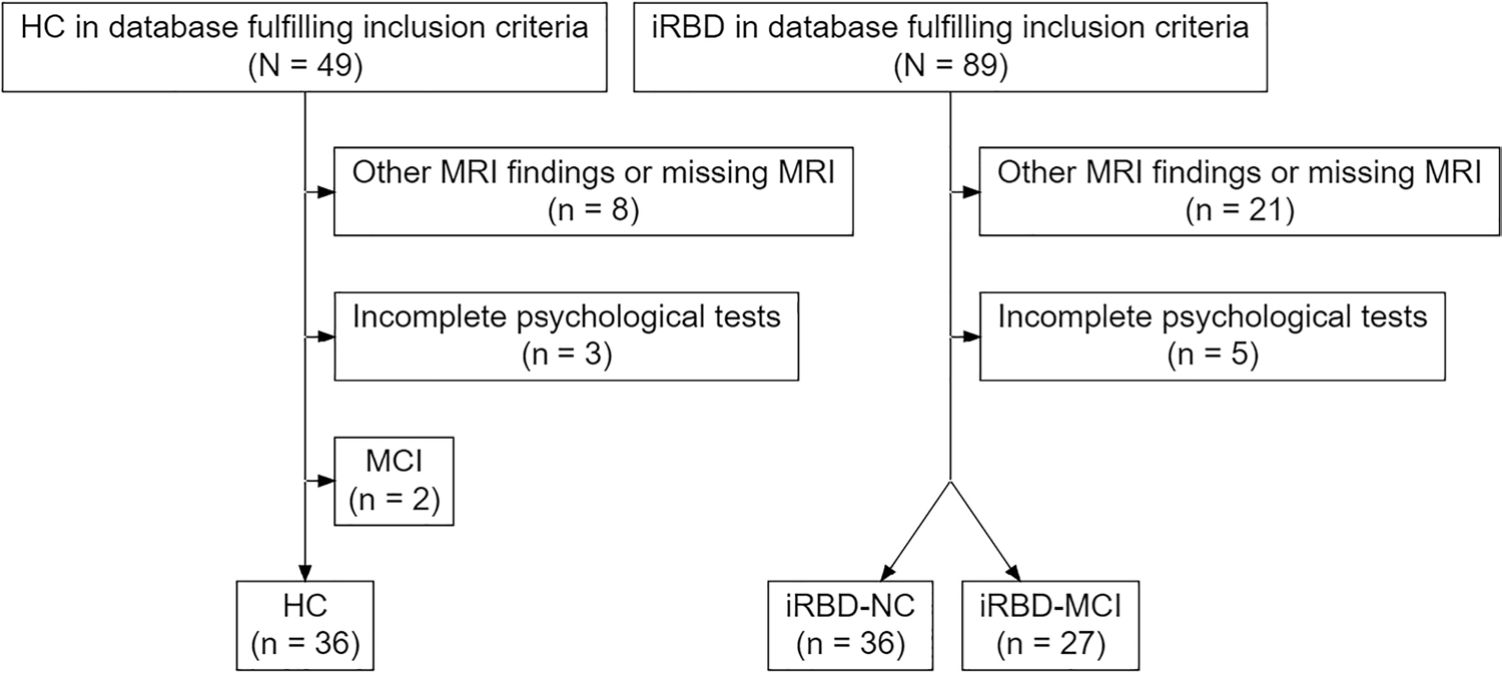
Flowchart of participant inclusion. Note. HC = healthy controls; iRBD = isolated rapid eye movement sleep behavior disorder; MCI = mild cognitive impairment; NC = normal cognition

A total of 89 iRBD patients and 49 HCs from our database met the inclusion criteria. Twenty-nine participants were excluded due to missing MRI data, motion artifacts, or other MRI abnormalities (e.g., confluent white matter hyperintensities), 8 for lacking more than 3 neuropsychological tests and 2 HCs for a diagnosis of MCI (Fig. 1). The final sample included 63 patients with iRBD and 36 HC. Twenty-seven iRBD patients (43%) met the criteria for MCI. HCs were slightly but significantly younger than the iRBD group (*p* = 0.045). There were no between-group differences in sex and education. iRBD were worse in global cognitive functioning as assessed using MoCA score (*p* < 0.003) and showed higher motor (*p* = 0.001), depressive (*p* < 0.001), and anxiety symptoms (*p* = 0.003) (Table 1).

**Table 1:** Socio-demographic and clinical characteristics of iRBD patients and controls

	HC^a^	iRBD^b^	iRBD-MCI^c^	iRBD-NC^d^	*p*^†^	*p*^††^	Post hoc
	(*n* = 36)	(*n* = 63)	(*n* = 27)	(*n* = 36)			
Age, years	63.76 (7.15)	66.73 (6.59)	65.09 (6.76)	67.96 (6.27)	0.045	0.030	a < d
Education, years	15.22 (3.27)	14.75 (3.02)	14.81 (3.05)	14.69 (3.03)	0.307	0.580	
Sex, male %	83	90	92	89	0.295	0.546	
Symptom duration, years	–	6.87 (7.90)	8.18 (10.07)	5.89 (5.74)	–	0.544^†††^	
MoCA	25.53 (2.01)	24.21 (2.57)	23.33 (2.48)	24.86 (2.46)	0.003	0.001	a > c; d > c
NART	120.58 (9.43)	118.21 (9.36)	115.30 (9.37)	120.39 (8.86)	0.235	0.048	
MDS-UPDRS-III	3.44 (4.36)	6.37 (5.49)	6.22 (5.72)	6.47 (5.40)	0.001	0.004	a > c; a > d
BDI-II	4.03 (3.10)	9.32 (7.53)	7.84 (5.84)	10.41 (8.48)	< 0.001	< 0.001	a > c; a > d
STAI-X1	31.33 (5.92)	36.71 (9.19)	35.88 (5.33)	37.32 (11.27)	0.003	0.010	a > c; a > d
STAI-X2	32.83 (6.61)	39.63 (8.97)	38.16 (7.22)	40.71 (10.03)	0.001	0.002	a > c; a > d
ESS	6.14 (3.80)	7.25 (4.40)	7.72 (4.94)	6.91 (4.01)	0.230	0.445	
SINBAR score^††††^	5.77 (2.80)	49.09 (24.35)	48.96 (29.47)	49.20 (19.74)	< 0.001	< 0.001	a < c; a < d
Tonic RWA index^††††^	0.93 (0.90)	18.60 (21.26)	20.67 (24.53)	16.91 (18.42)	< 0.001	< 0.001	a < c; a < d
Phasic RWA index^††††^	3.77 (2.26)	25.21 (16.80)	24.78 (17.88)	25.57 (16.15)	< 0.001	< 0.001	a < c; a < d
Mixed RWA index^††††^	0.40 (0.53)	10.29 (15.32)	10.37 (12.91)	10.22 (17.24)	< 0.001	< 0.001	a < c; a < d

*HC* healthy controls, *iRBD* isolated rapid eye movement sleep behavior disorder, *MCI* mild cognitive impairment, *NC* normal cognition, *Symptom duration* time between the subjective onset of iRBD symptoms and assessment, *MoCA* Montreal Cognitive Assessment Czech version, *NART* National Adult Reading Test, *MDS-UPDRS-III* Movement Disorders Society-Unified Parkinson’s Disease Rating Scale, part III, *BDI-II* Beck Depression Inventory, Second Edition, *STAI* State-Trait Anxiety Inventory (state anxiety, X1) and (trait anxiety, X2), *ESS* Epworth Sleepiness Scale, *RWA* REM without atonia

^†^HC vs iRBD

^††^HC vs iRBD-NC vs iRBD-MCI

^†††^iRBD-NC vs iRBD-MCI

^††††^iRBD (*n* = 58), iRBD-MCI (*n* = 26), iRBD-NC (*n* = 32)

### MRI differences between groups

VBM, DBM and the comparison of ROI volumes segmented by Hammers Atlas yielded no significant between-group differences. There was a statistical trend for reduced volumes of several ROIs in the RBD groups; compared to HC, RBD-NC patients had lower volumes of the right cerebellum while RBD-MCI patients had lower volumes in the cuneus, cerebellum, lingual gyrus, putamen, nucleus accumbens and parts of parietal and temporal lobes (*p* < 0.05 uncorrected; Supplemental Table 2).

### Neuropsychological assessment

Significant between-group differences were found in measures of episodic memory (MIST – time-based, MBT – Total Delayed Free Recall), attention/working memory (TMT-A), executive functions (PST – interference, TMT-B), processing speed (SDMT, PST – Dots); and language (VF – letter K). Except for MIST (time-based), MBT (Total Delayed Free Recall), and PST (interference), in which post hoc tests revealed no significant differences, iRBD-MCI performed worse than HC and iRBD-NC groups on all the above cognitive tests. For other neuropsychological variables, no significant differences were detected. Notably, no significant differences were found between HC and iRBD-NC groups (Table 2).

**Table 2:** Group differences in neuropsychological tests

		*df*	*F*	η_p_^2^	*p*^†^	Tukey HSD
Episodic memory	RAVLT (Total recall 1–5)					
	group	1	1.988	0.020	0.162	
	MCI status	1	6.426	0.063	0.013	
	RAVLT (Trial 6)					
	group	1	0.969	0.010	0.327	
	MCI status	1	0.633	0.007	0.428	
	RAVLT (delayed recall)					
	group	1	2.650	0.027	0.107	
	MCI status	1	6.806	0.066	0.011	
	RAVLT (recognition)					
	group	1	3.338	0.034	0.071	
	MCI status	1	5.778	0.057	0.018	
	MIST (event-based)					
	group	1	2.543	0.026	0.114	
	MCI status	1	0.079	0.001	0.779	
	MIST (time-based)					
	group	1	0.014	< 0.001	0.907	
	MCI status	1	8.123	0.078	**0.005**	ns
	MBT (Total Cued Recall)					
	group	1	0.093	0.001	0.761	
	MCI status	1	1.378	0.014	0.243	
	MBT (Paired Recall Pairs)					
	group	1	0.104	0.001	0.747	
	MCI status	1	1.302	0.013	0.257	
	MBT (Total Delayed Paired Recall)					
	group	1	0.021	< 0.001	0.885	
	MCI status	1	2.449	0.025	0.121	
	MBT (Delayed Paired Recall Pairs)					
	group	1	0.003	< 0.001	0.956	
	MCI status	1	0.926	0.010	0.338	
	MBT (Total Free Recall)					
	group	1	0.246	0.003	0.621	
	MCI status	1	2.097	0.021	0.151	
	MBT (Total Delayed Free Recall)					
	group	1	0.129	0.001	0.720	
	MCI status	1	7.743	0.075	**0.006**	ns
Attention / working memory	LNS					
	group	1	0.050	0.001	0.824	
	MCI status	1	3.426	0.034	0.067	
	TMT-A					
	group	1	0.562	0.006	0.455	
	MCI status	1	25.699	0.211	** < 0.001**	HC > iRBD-MCI; iRBD-MCI < iRBD-NC
Executive functions	PST (colors)					
	group	1	0.020	< 0.001	0.888	
	MCI status	1	4.213	0.042	0.043	
	PST (interference)					
	group	1	0.139	0.001	0.710	
	MCI status	1	7.553	0.073	**0.007**	ns
	TMT-B					
	group	1	1.189	0.012	0.278	
	MCI status	1	21.29	0.182	** < 0.001**	HC > iRBD-MCI; iRBD-MCI < iRBD-NC
	VF (animals/clothes)					
	group	1	0.524	0.005	0.471	
	MCI status	1	2.872	0.029	0.093	
Visuospatial functions	CDT					
	group	1	0.022	< 0.001	0.881	
	MCI status	1	6.237	0.061	0.014	
	MoCA (cube)					
	group	1	2.923	0.030	0.091	
	MCI status	1	2.450	0.025	0.121	
Processing speed / psychomotor speed	GPT (left hand)					
	group	1	1.146	0.012	0.287	
	MCI status	1	4.830	0.048	0.030	
	GPT (right hand)					
	group	1	0.797	0.008	0.374	
	MCI status	1	3.997	0.040	0.048	
	SDMT					
	group	1	3.469	0.035	0.066	
	MCI status	1	10.088	0.095	**0.002**	HC > iRBD-MCI; iRBD-MCI < iRBD-NC
	PST (Dots)					
	group	1	1.460	0.015	0.230	
	MCI status	1	8.625	0.082	**0.004**	iRBD-MCI < iRBD-NC
	PST (words)					
	group	1	0.984	0.010	0.324	
	MCI status	1	4.236	0.042	0.042	
Language	VF (K)					
	group	1	1.963	0.020	0.164	
	MCI status	1	10.767	0.101	**0.001**	iRBD-MCI < iRBD-NC
	VF (action verb)					
	group	1	0.009	< 0.001	0.926	
	MCI status	1	0.527	0.005	0.470	
	VF (vegetables)					
	group	1	0.736	0.008	0.393	
	MCI status	1	4.269	0.043	0.042	

Summary of the nested ANOVAs with fixed factors group (iRBD, HC) and MCI status (NC, MCI) on demographically adjusted *z*-scores. *df* degrees of freedom, *F F* statistic, η_p_^2^ partial eta squared. *HC* healthy controls, *iRBD* isolated rapid eye movement sleep behavior disorder, *MCI* mild cognitive impairment, *NC* normal cognition, *ns* not significant post hoc test, *RAVLT* Rey Auditory Verbal Learning Test, *MBT* Memory Binding Test, *MIST* Memory for Intentions Screening Test, *TMT* Trial Making Test, *LNS* Letter-Number Sequencing from Wechsler Adult Intelligence Scale, Third Revision, *PST* Prague Stroop Test, *VF* Verbal fluency, *CDT* Clock Drawing Test, *GPT* Grooved Pegboard Test, *MoCA* Montreal Cognitive Assessment, *SDMT* Symbol Digit Modalities Test

†*p* values < 0.05 after Benjamini–Hochberg correction are in bold

In the PCA analysis, a six-component model with eigenvalues greater than 1 was selected (Supplemental Table 3). Patients with iRBD-MCI scored worse than HC, especially in the third (episodic memory) and second (processing speed/executive functions) components, however, these differences were not statistically significant after correction for multiple comparisons (Table 3).

**Table 3:** Group differences in cognitive domains

	*df*	*F*	η_p_^2^	*p*^†^
Associative memory				
group	1	0.115	0.001	0.735
MCI status	1	1.070	0.011	0.303
Processing speed/Executive functions				
group	1	0.568	0.006	0.453
MCI status	1	4.145	0.041	0.045
Episodic memory				
group	1	5.931	0.058	0.017
MCI status	1	1.158	0.012	0.285
Psychomotor speed				
group	1	0.018	< 0.001	0.893
MCI status	1	1.031	0.011	0.312
Language				
group	1	0.006	< 0.001	0.939
MCI status	1	0.570	0.006	0.452
Visuospatial functions				
group	1	3.863	0.039	0.052
MCI status	1	2.742	0.028	0.101

Summary of the nested ANOVAs with fixed factors group (iRBD, HC) and MCI status (with, without) on scores derived from the principal component analysis. *df* degrees of freedom, *F F* statistic, η_p_^2^ partial eta squared

†No domain is significant after Benjamini–Hochberg correction

### Correlations between cognitive tests and brain morphometry in iRBD patients

**Fig. 2:**
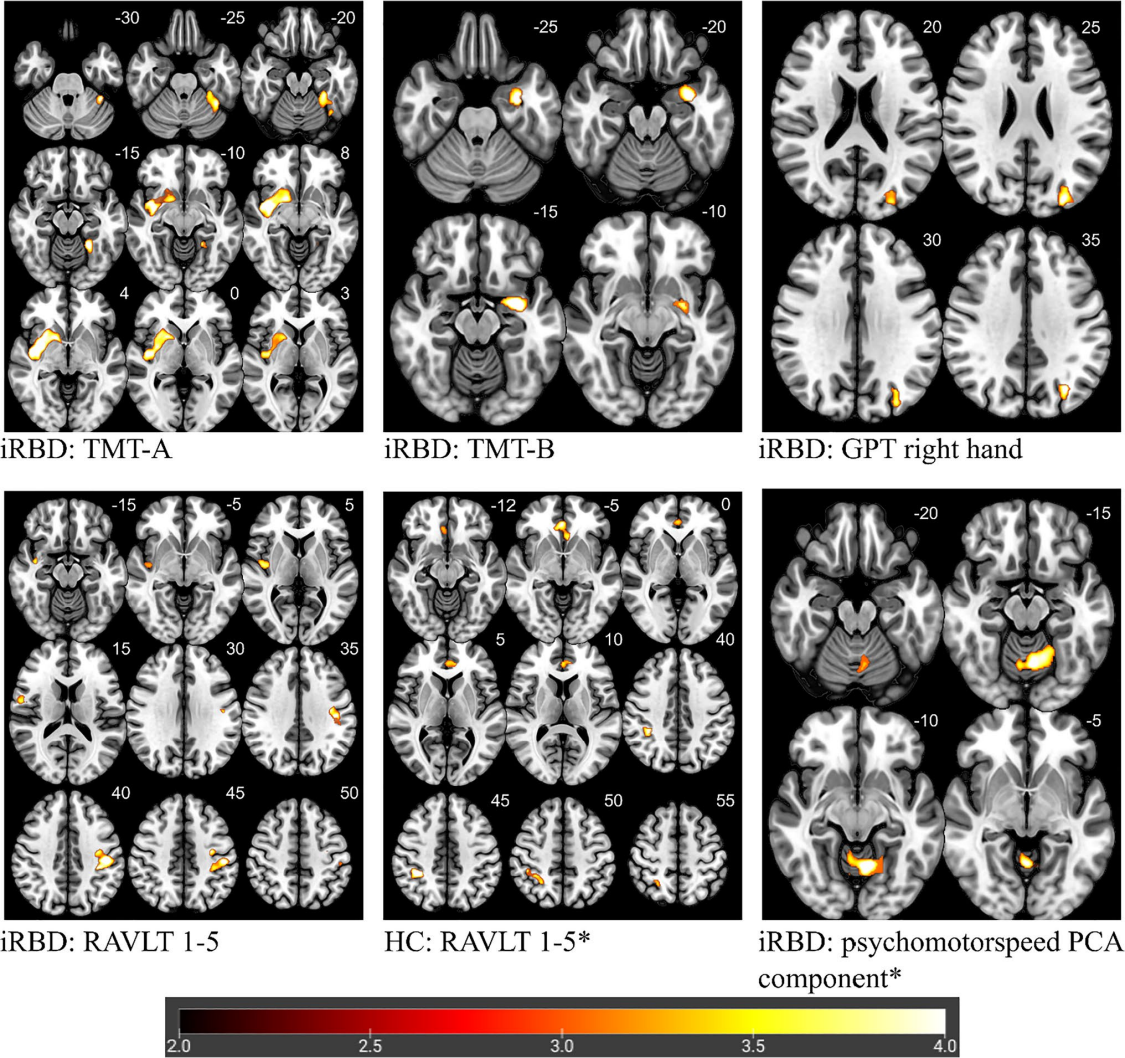
Correlation between performance in cognitive tests and brain morphology analyzed with VBM. *Note.* Highlighted significant clusters thresholded at p < 0.05 at cluster level, corrected for family wise error. Color scale represents decimal logarithm of p-level. Z-coordinates in the Montreal Neurologic Institute space (in millimeters) are indicated next to each slice (top right). Lower values of Psychomotor speed (PCA component) indicate better cognitive performance. HC = healthy controls; iRBD = isolated rapid eye movement sleep behavior disorder; TMT-A = Trail Making Test, part A; TMT-B = Trail Making Test, part B; GPT = Grooved Pegboard Test; RAVLT 1-5 = Rey Auditory Verbal Learning Test 1-5, PCA = principal components analysis. * indicating negative correlation

Using VBM, the performance in TMT-A, TMT-B, GPT right hand, RAVLT 1–5 and the PCA component psychomotor speed were identified to significantly correlate with brain morphology in iRBD patients (Fig. 2; Supplemental Table 4).

TMT-A performance correlated positively with a cluster in the right hemisphere including regions of the putamen, insula and temporal superior lobe (*p* < 0.001) and a second cluster in the left cerebellar hemisphere (*p* = 0.026). TMT-B performance correlated positively with clusters in the left hemisphere including the amygdala, hippocampus and parahippocampal gyrus (*p* = 0.050). Right-hand GPT correlated positively with a cluster containing regions in the occipital and parietal lobe of the left hemisphere (*p* = 0.043). A cluster containing parts of the precentral, postcentral and supramarginal gyrus of the left hemisphere (*p* = 0.038) as well as another cluster in the right hemisphere containing parts of the insula, hippocampus and temporal lobe correlated positively with RAVLT 1–5 (*p* = 0.023). The PCA component psychomotor speed showed a significant negative correlation with regions in the rostral vermis and adjacent parts of both cerebellar hemispheres (*p* = 0.010).

In HC no correlations between brain morphology and the results of TMT-A, TMT-B, GPT right hand and psychomotor speed were detectable. Correlation analysis for RAVLT 1–5 in HC showed one significant cluster, which was located in different brain regions and had a different direction (negative) than correlations in iRBD patients. The cluster is in the cingulum and frontal lobe of both hemispheres (*p* = 0.020).

Complementary ROI-based analysis revealed significant correlations between relative volumes of grey matter and the performance in TMT-A, GPT left and right hand as well as with PCA component psychomotor speed in iRBD, which were not observed in HC. The performance of RAVLT 1–5 correlated with the relative sizes of several brain regions (e.g., pallidum, nucleus accumbens, insula) in iRBD from which some also correlated in HC (Table 4).

**Table 4:** ROI-based analysis of the correlation between performance in cognitive tests and brain regional volumes segmented by Hammers atlas

	iRBD		HC	
	*r*	*p*	*r*	*p*
TMT-A				
Right Putamen*	0.407	0.001	-0.033	0.852
Right Insula*	0.366	0.003	-0.123	0.482
Right Nucleus Accumbens*	0.364	0.003	0.012	0.943
Left Nucleus Accumbens*	0.364	0.004	-0.062	0.725
Left Putamen*	0.365	0.004	-0.074	0.671
Left Precentral Gyrus*	0.330	0.009	-0.176	0.311
Left Insula*	0.311	0.014	-0.130	0.456
GPT (left hand)				
Right Insula*	0.434	< 0.001	-0.174	0.318
GPT (right hand)				
Right Insula*	0.314	0.013	-0.265	0.124
RAVLT 1–5				
Right Pallidum *	0.469	< 0.001	0.015	0.931
Left Pallidum*	0.404	0.001	0.113	0.518
Left Nucleus Accumbens*	0.399	0.001	-0.262	0.128
Right Insula*	0.402	0.001	-0.272	0.115
Left Brainstem*	0.354	0.002	-0.279	0.105
Right Superior Parietal Gyrus	0.376	0.003	-0.369	0.029
Right Brainstem*	0.373	0.003	-0.249	0.149
Right Nucleus Accumbens	0.357	0.004	-0.480	0.004
Right Precentral Gyrus	0.362	0.004	-0.541	0.001
Left Insula*	0.352	0.005	-0.274	0.112
Left Nucleus Caudate*	0.354	0.005	-0.254	0.141
Right Nucleus Caudate*	0.348	0.006	-0.230	0.184
Left Inferior Lateral Parietal Lobe	0.343	0.006	-0.345	0.043
Left Precentral Gyrus	0.335	0.008	-0.348	0.040
Right Posterior Temporal Lobe*	0.328	0.009	-0.219	0.206
Right Cuneus*	0.327	0.010	0.199	0.253
Left Postcentral Gyrus	0.327	0.010	-0.552	0.001
Right Lingual Gyrus*	0.322	0.011	0.149	0.349
Right Inferior Lateral Parietal Lobe*	0.307	0.015	-0.237	0.170
Right Fusiform Gyrus*	0.295	0.020	-0.064	0.717
Left Posterior Temporal Lobe*	0.293	0.021	-0.198	0.255
Left Superior Parietal Gyrus*	0.274	0.031	-0.140	0.424
Psychomotor speed (PCA component)				
Left Cerebellum*	-0.390	0.002	-0.043	0.804
Left Anterior Temporal Lobe, Medial Part*	-0.360	0.004	-0.101	0.562
Left Insula*	-0.310	0.014	0.104	0.551

Pearson correlation coefficients adjusted for age for all significant associations in RBD subjects are shown (*p* < 0.05, FDR corrected). Corresponding correlation coefficients *r* and *p* values in HC are shown for comparison. Lower values of Psychomotor speed (PCA component) indicate better cognitive performance. *iRBD* Isolated rapid eye movement sleep behavior disorder, *HC* healthy controls, *TMT-A* Trail Making Test, part A, *GPT* Grooved Pegboard Test, *RAVLT 1–5* Rey Auditory Verbal Learning Test 1–5, *PCA* principal components analysis

*Significant correlation in iRBD but not in HC

**Fig. 3:**
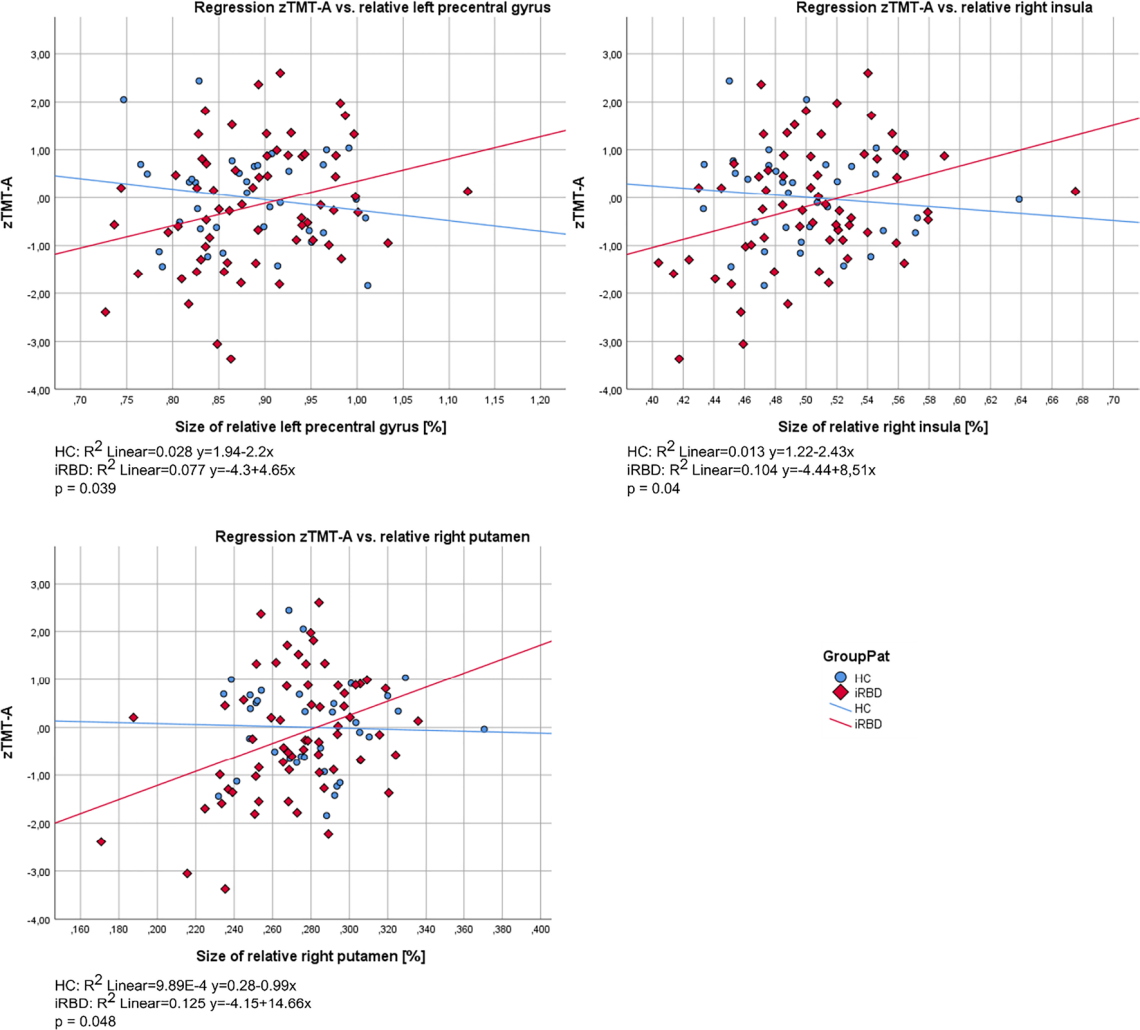
Linear regression plots of brain regional volumes associated with the performance of TMT-A. *Note. p* value for difference of regressions slopes between iRBD and HC is shown in every graph. HC = healthy controls; iRBD = isolated rapid eye movement sleep behavior disorder; z = z-score; TMT-A = Trail Making Test, part A

When regression slopes of cognitive performance versus regional brain volumes analyses were statistically compared in iRBD and HC, only TMT-A showed a significant between-group difference. Linear regression slope differences between iRBD and HC for TMT-A were observed in the left precentral gyrus (*p* < 0.039), right insula (*p* < 0.040), and right putamen (*p* < 0.048; Fig. 3).

**Fig. 4:**
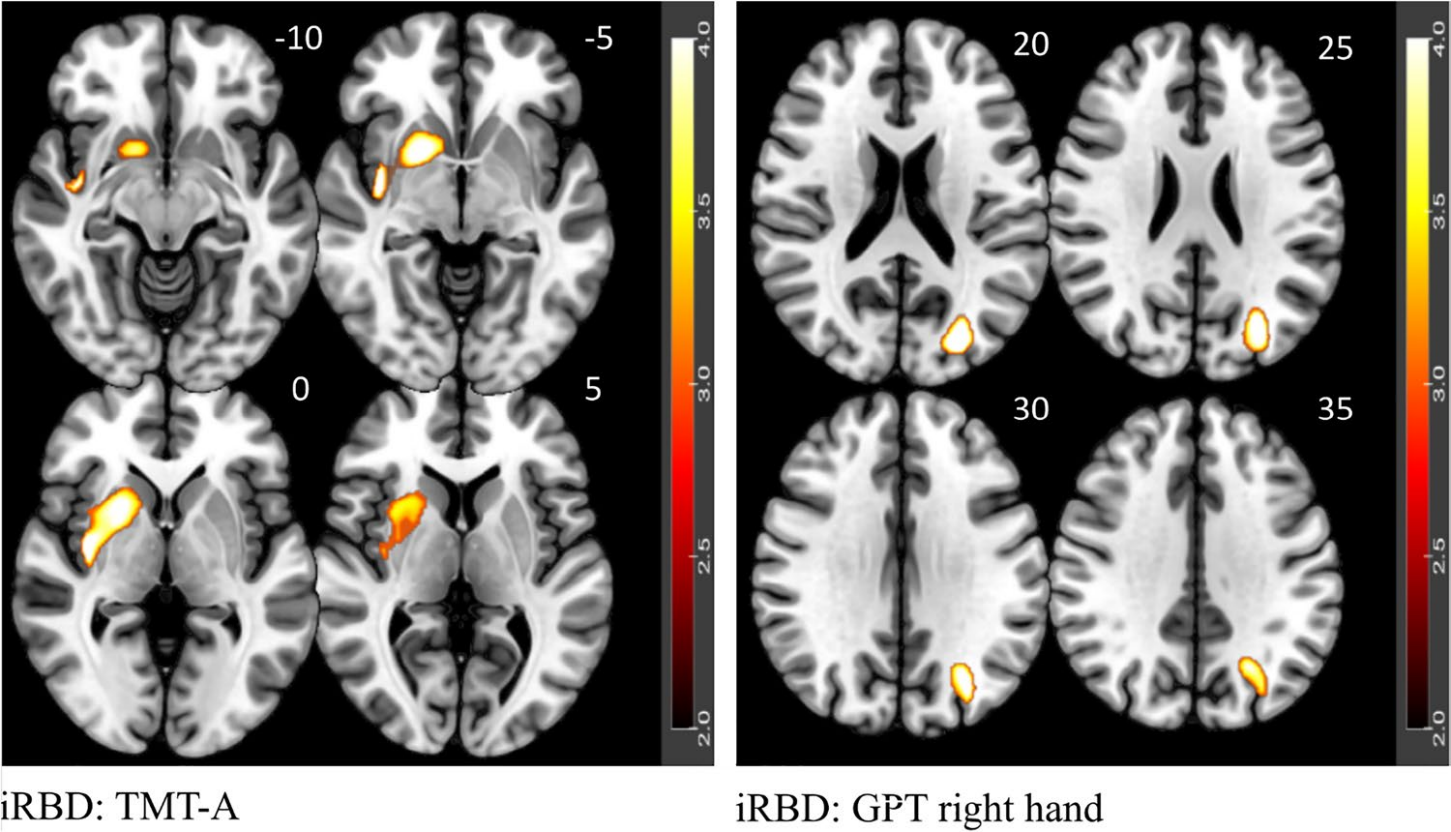
Correlation between performance in cognitive tests and brain morphology analyzed with DBM. Note. Highlighted significant clusters thresholded at p < 0.05 at cluster level, corrected for family wise error. Color scale represents decimal logarithm of p-level. Z-coordinates in the Montreal Neurologic Institute space (in millimeters) are indicated next to each slice (top right). iRBD = isolated rapid eye movement sleep behavior disorder; TMT-A = Trail Making Test, part A; GPT = Grooved Pegboard Test

The DBM revealed a significant correlation between TMT-A and a cluster involving putamen, insula, pallidum, caudate and superior temporal lobe (*p* = 0.004). For the GPT right hand a correlation was found with a cluster involving the middle and superior occipital lobe and the superior parietal lobe (*p* = 0.017)(Fig. 4 and Supplemental Table 5).

### Correlations between cognitive tests and REM sleep without atonia severity

Correlation analysis of psychological variables with RWA indexes in the subgroup where all data were available (58 iRBD patients) showed no significant associations. However, there was some tendency towards worse scoring, primarily in TMT-B and memory measures, with higher RWA (Supplemental Table 6).

## Discussion

Very few studies have been published on the cognitive deficit and underlying neuroanatomical mechanism in iRBD, and with inconclusive results. These inconclusive results may be due to methodological issues such as small sample size, use of a single analysis technique, or ignoring MCI status. Bearing this in mind, VBM, DBM, and ROI analyses and complex neuropsychological battery were used in this study to better characterize the possible underlying neuroanatomical mechanism of cognitive dysfunction.

### MRI differences between groups

There were no significant differences between iRBD and HC in terms of brain morphological changes. These findings reflect the current inconclusive results of studies using VBM in iRBD [8, 28]. As suggested in other papers [28], this may be due to a heterogeneous sample composed of patients at a very early stage, who may convert to different neurodegenerative diseases with pathological synuclein storage. Based on the results presented, we can also assume that the underlying pathology has not yet affected our sample enough to be detectable by VBM, DBM, or ROI analyses. The median time between the subjective onset of iRBD symptoms and assessment was 5 years. This explanation is also supported by the results of the ROI analysis without correction for multiple comparisons, which showed a statistical trend of regional atrophy in iRBD (Supplemental Table 2) with a similar posterior pattern reported in other studies [4, 7].

Splitting the sample according to the presence of MCI did not yield any further significant results as in the case of Remillard-Pelchat et al. [29], who identified differences in the insula, middle and superior temporal, angular and orbitofrontal gyrus. The presence of MCI is usually associated with worse disease progression and more profound morphological changes in both iRBD and PD [5, 30]. The failure to detect differences in this study could suggest that the manner in which MCI is diagnosed is of great importance, as the criteria and methods vary between studies [5, 29]. Furthermore, the detectability of morphological changes by VBM, ROI or DBM could be sensitive only at later stages of the disease. Both of the above studies used a sample with symptom duration longer than 11 years. Which is almost twice as long as in our iRBD-NC group and three years longer than in iRBD-MCI group.

### Neuropsychological assessment

iRBD did not differ from HC in any of the psychological variables, probably for the same reasons as in the morphometric analyses. However, when taking into account the MCI status, iRBD-MCI scored lower than the other groups in TMT-A, TMT-B, SDMT, VF (letter K), and PST (dots). Despite the different interpretations of each test in terms of cognitive function, all tests share a strong processing speed or motor speed component, which might suggest a general slowness of iRBD-MCI. Several studies have already reported lower scores in processing/motor speed [7, 11], and this also fits well with the observed gradual deterioration in psychomotor speed in PD [31]. Why a predominantly reported executive deficit was not observed may result, among other things, from differences in the constructs measured depending on disease progression. For example, Koerts et al. [32] show in verbal fluency that this test measures psychomotor speed in the early stages of PD, and only in the later stages, it measures executive functions. Our study focuses on patients before the development of overt neurological disease, which would be consistent with the findings of Koerts et al. [32]. The classification of the tests into different cognitive domains and the classification of the domains themselves may also play a role. Although the meta-analytic study by Leitner et al. [2] confirmed the significance of deficits in executive functions, it also highlighted considerable inconsistency in their measurement. Moreover, when dividing executive functions into several components, they found, as in this study, processing speed as the most affected.

### Correlations between cognitive tests and brain morphometry

The stratification into iRBD and HC yielded several significant results compared to the stratification based on the MCI status. Using VBM, we identified clusters corresponding to the performance of TMT-A, TMT-B, GPT (right hand), RAVLT 1–5, and psychomotor speed (PCA component). These correlations were unique to iRBD, as they were not replicable in HC and thus presumably reflect disease-specific abnormalities.

Specifically, VBM revealed a correlation of TMT-A with two clusters, one in the cortical and subcortical region including the striatum, insula, temporal superior lobe, pallidum and amygdala and the second in the cerebellar region (Fig. 2, Supplemental Table 4). A correlation with a very similar cluster to the former was found in DBM as well (Fig. 4, Supplemental Table 5). This indicates not only atrophy but also significant changes in the shape of these brain regions, which are typically associated with motor control, executive, visuospatial functions and psychomotor speed deficits [6, 33, 34].

The TMT-A is designed to measure psychomotor speed, and performance in this cognitive domain have been associated mostly with basal ganglia in alpha-synucleinopathies [33, 34]. Thus, the cognitive domains and anatomical structures found in this study overlap considerably with those previously reported. If we also consider previously published results examining brain or TMT-A differences in iRBD [6, 7, 28, 35], this study fits into the overall picture of the results published so far and confirms the role of the striatum, insula, cerebellum and pallidum in the deficits of psychomotor speed and visual attention as measured by TMT-A.

The cluster correlating with performance in TMT-B mainly consisted of parts of the limbic system, specifically the amygdala, hippocampus, parahippocampal and fusiform gyrus. Significant differences between HC and iRBD are mostly reported in the TMT-B rather than the TMT-A and are referred to as indicators of deficits in executive functions [2, 36]. This interpretation is based on the nature of the test design [37] and also that TMT-B scores are associated with frontal regions [38] rather than temporal regions as found in this study or Zakzanis, Mraz, Graham [39]. However, based on the correlations presented, it seems that the lower performance in TMT-B in iRBD-MCI may be driven by changes in structures related to memory deficits that are common in iRBD [2]. In the absence of functional imaging, this relationship is unclear, and this issue needs to be addressed in future studies. Nevertheless, the greater involvement of memory in the TMT-B is not illogical, as it is necessary to recall the next letter of the alphabet while remembering the last number/letter as one progresses through this test. In view of the above, the interpretation of the TMT-B in iRBD studies as a purely executive test related to frontal lobe disruption should be carefully considered for the time being.

The GPT (right hand) showed a significant correlation with the left occipital and parietal regions. The cluster covering the same regions was also correlated in DBM. The absence of correlations with structures responsible for motor control suggests that sensorimotor respectively visuomotor integrative functions mediated by the occipital and parietal lobes are likely to play a more important role in GPT performance, as also suggested by others [40]. Indeed, the poorer performance of iRBD patients in visuospatial abilities associated with parietal regions has also been reported by Pereira et al. [4] or Rahayel et al. [6]. The overall picture is also reinforced by the study of Campabadal et al. [41], which identified impairments in functional connectivity between temporal and parietal regions that correlated with tests of processing speed, arguing that lower performance on these tests may actually be due to impaired integration of visuospatial processing and visuo-verbal decodification mediated by ventral and dorsal stream. Thus, there are strong indications that parietal and occipital regions are responsible for deficits in iRBD in visuomotor integration as measured by GPT. The same is shown in another correlation analysis performed on the PCA component psychomotor speed, loaded mainly by GPT, with the lingual gyrus and cerebellum. PCA allows a cleaner measurement of basic cognitive functions through latent variable extraction. The use of latent variables highlighted the importance of the lingual gyrus in particular among all correlations with the occipital and parietal regions found purely for GPT. Yet, given the absence of between-group differences in mean scores both with and without taking into account the MCI status, see this study and others [42, 43], the question arises whether GPT is sensitive enough to provide any clinically useful information.

The correlations of RAVLT 1–5 primarily with the parietal and temporal regions stand out. The RAVLT 1–5 is the only test that does not include a distinct motor or psychomotor speed component and measures learning capacity and short-term memory during the learning phase. Deficits in this domain are the second most frequently reported after executive functions [1, 2]. And disruptions in the parietal and temporal regions are equally well described [4, 36]. Even a direct link between verbal learning and memory in iRBD with disruptions in temporal, occipital and insular regions has been described several times [6, 36]. All this suggests a systematic difficulty of iRBD with memory and associated morphological changes primarily in temporal and parietal regions. Furthermore, it complements well the findings in the case of GPT and supports the hypothesis of impaired integration of visuospatial processing and visuo-verbal decodification on the anatomical basis of posterior regions.

Overall, both parts of the TMT, especially part A, appear to be robust in terms of correlations with brain morphology and differences in means between groups. However, the findings for the other variables are not as clear-cut. HC, iRBD-NC and iRBD-MCI differed in SDMT, PST (Dots), and VF (latter K), which did not correlate with any brain region. While GPT (right hand), RAVLT 1–5, and psychomotor speed (PCA component) were correlated, iRBD did not differ from HC in these variables. This discrepancy is not easily explained. One reason may be the somewhat artificial division into groups with or without MCI according to a single cut-off score, in this case, a -1.5 *z*-score.

Regarding the secondary objective of testing the association of RWA with cognitive tests, we found no significant results, which was also the case when the analysis was performed only on the iRBD-MCI subgroup. Although these results are not in line with others [9], they clearly support hypotheses stated above, that some of physiological and morphological changes could not be detected by standard techniques in early stages of the disease. As mentioned above, the duration of iRBD symptoms in this study is fundamentally shorter, which is also true for the severity of RWA. For example, Figorilli et al. [9] found an association in a sample that had SINBAR scores ranging from 49.9 to 81.6, the minimum of which essentially corresponds to the median of 49.30 found in this study. Thus, inherently, some association between cognition and RWA severity can be assumed, but this cannot be observed in patients with iRBD in the early stages by conventional methods.

### Limitations

This study has several limitations that should be clearly stated. There are currently no standardized diagnostic criteria for MCI in patients with RBD. Therefore, a neuropsychological battery consisting of tests of attention/working memory, executive functions, language, episodic memory, visuospatial functions and processing speed/psychomotor speed was used to cover most of the relevant domains. The same applies to the cut-off value for MCI. Thus, the cut-off value of -1.5 *z-*score commonly reported for PD was used [44]. This study is exploratory in nature with a cross-sectional design. Thus, it remains a matter of interest, for example, whether the same cognitive deficit may be due to impairment of other structures as the disease progresses, or whether the primary impairment is in processing speed in general or just motor speed specifically. In five patients (7%) with iRBD, the RWA indexes could not be traced in the database, but these missing data represent a negligible amount that, in our opinion, has no impact on the overall outcome. Finally, depression and anxiety were not included as covariates in the analyses due to the lack of correlations with other variables and in order to measure the overall effect of iRBD.

## Conclusion

This study provides consistent results that show profound deficits in motor/psychomotor/processing speed and learning that correlate with brain volume in iRBD. Although morphological changes could not be directly detected in our sample using VBM, DBM or ROI analysis, the results before statistical correction point to the same posterior regions that have already been associated with iRBD. Overall, the most robust finding is the deficit in psychomotor speed and visual attention measured by TMT-A associated with reduced volume of the striatum, insula, cerebellum, temporal lobe, pallidum and amygdala.

### Supplementary Information

Below is the link to the electronic supplementary material.

Supplementary file1 (DOCX 42 KB)

## Data Availability

The data that support the findings of this study are available on request from the corresponding author. The data are not publicly available due to privacy or ethical restrictions.
