# Supplementary material for: Cortical and subcortical morphometric changes and their relation to cognitive impairment in isolated REM sleep behavior disorder
Source: Neurol Sci. 2023 Sep 5;45(2):613–27. doi: 10.1007/s10072-023-07040-z (PMC10791856; doi:10.1007/s10072-023-07040-z)
Supplement: Supplementary file 1 — Supplementary file1 (DOCX 42 KB) [file 10072_2023_7040_MOESM1_ESM.docx]

**Supplemental Table 1**Description of neuropsychological variables

| Domain | Variable | Description |
| --- | --- | --- |
| Episodic memory | RAVLT (Total recall 1-5) | Total sum of correctly recalled words in five trials. |
|  | RAVLT (Trial 6) | Recalled words from the first list after the List B trial. |
|  | RAVLT (delayed recall) | Recalled words from the first list after 30 min. |
|  | RAVLT (recognition) | Recognition after the delayed recall. |
|  | MIST (event-based) | Prospective memory based on event cues. |
|  | MIST (time-based) | Prospective memory based on time cues. |
|  | MBT (Total Cued Recall) | Total words recalled with the provided clue. |
|  | MBT (Paired Recall Pairs) | Total pairs of words recalled. |
|  | MBT (Total Delayed Paired Recall) | Total words recalled with the provided clue after 30 min. |
|  | MBT (Delayed Paired Recall Pairs) | Total pairs of words recalled after 30 min. |
|  | MBT (Total Free Recall) | Total words recalled. |
|  | MBT (Total Delayed Free Recall) | Total words recalled after 30 min. |
| Attention / working memory | LNS | Number correctly ordered letters and numbers so that the numbers come first and then the letters. |
|  | TMT-A | Time in seconds required to complete TMT Part A. |
| Executive functions | PST (colors) | The time in seconds required to name colors printed with a different color name. |
|  | PST (interference) | The time in seconds required to name colors printed with a different or equivalent color name. |
|  | TMT-B | Time in seconds required to complete TMT Part B |
|  | VF (animals/clothes) | Total named words from the two switching categories (animals/clothes). |
| Visuospatial functions | CDT | Total CDT score based on Royall's scoring system. |
|  | MoCA (cube) | MoCA Cube subtest score. |
| Processing speed / psychomotor speed | GPT (left hand) | Total time required to complete the GPT with the left hand. |
|  | GPT (right hand) | Total time required to complete the GPT with the right hand. |
|  | SDMT | The total number of numbers correctly matched to the symbols according to the key. |
|  | PST (Dots) | The time in seconds required to name colors. |
|  | PST (words) | The time in seconds required to name the colors printed with meaningful words. |
| Language | VF (K) | Total named words beginning with the letter “K”. |
|  | VF (action verb) | Total named words from the category “action verb”. |
|  | VF (vegetables) | Total named words from the category “vegetables”. |

*Note.* RAVLT = Rey Auditory Verbal Learning Test; MBT = Memory Binding Test; MIST = Memory for Intentions Screening Test; TMT = Trial Making Test; LNS = Letter-Number Sequencing from Wechsler Adult Intelligence Scale, Third Revision; PST = Prague Stroop Test; VF = Verbal fluency; CDT = Clock Drawing Test; GPT = Grooved Pegboard Test; MoCA = Montreal Cognitive Assessment; SDMT = Symbol Digit Modalities Test.

**Supplemental Table 2**
ROI volumes defined by Hammers atlas with a statistical trend for significant between group differences

|  |  | T-value | Z-value | *p* |
| --- | --- | --- | --- | --- |
| HC > iRBD total | |  |  |  |
|  | left Cuneus | 2.36 | 2.32 | 0.010 |
|  | left Cerebellum | 2.01 | 1.98 | 0.024 |
|  | right Cerebellum | 2.11 | 2.08 | 0.019 |
|  | left Inferior Lateral Parietal Lobe | 1.83 | 1.80 | 0.035 |
|  | right Lingual Gyrus | 2.19 | 2.16 | 0.015 |
|  | right Lateral Occipital Lobe | 1.87 | 1.85 | 0.032 |
| HC > iRBD-NC | |  |  |  |
|  | Right Cerebellum | 1.71 | 1.69 | 0.046 |
| HC > iRBD-MCI | |  |  |  |
|  | Left Cuneus | 2.43 | 2.36 | 0.009 |
|  | Left Superior Parietal Gyrus | 2.29 | 2.23 | 0.013 |
|  | Inferior Lateral Parietal Lobe | 1.90 | 1.86 | 0.031 |
|  | Nuccleus Accubens | 1.88 | 1.84 | 0.033 |
|  | Left Anterior Medial Temporal Lobe | 1.81 | 1.78 | 0.037 |
|  | Left Cerebellum | 1.78 | 1.75 | 0.040 |
|  | Right Cerebellum | 1.82 | 1.79 | 0.037 |
|  | Left Putamen | 1.77 | 1.74 | 0.041 |
|  | Right Lingual Gyrus | 2.66 | 2.58 | 0.005 |

*Note.* *p* values uncorrected. HC = healthy controls; iRBD = isolated rapid eye movement sleep behavior disorder; MCI = mild cognitive impairment; NC = normal cognition.

**Supplemental Table 3**
Principal components analysis: factor loadings of 28 neuropsychological test scores on six components, rotated and extracted with varimax rotation

|  | AM | PS/E | EM | PS | L | VisF |
| --- | --- | --- | --- | --- | --- | --- |
| MBT (Total Cued Recall) | **0.92** | -0.16 | 0.16 | -0.01 | 0.13 | 0.11 |
| MBT (Paired Recall Pairs) | **0.92** | -0.14 | 0.21 | 0.00 | 0.10 | 0.09 |
| MBT (Total Delayed Paired Recall) | **0.94** | -0.09 | 0.16 | -0.03 | 0.12 | 0.05 |
| MBT (Delayed Paired Recall Pairs) | **0.95** | -0.07 | 0.14 | -0.03 | 0.08 | 0.04 |
| MBT (Total Free Recall) | **0.70** | -0.09 | 0.18 | 0.01 | 0.31 | 0.09 |
| MBT (Total Delayed Free Recall) | **0.59** | 0.01 | 0.25 | -0.04 | 0.38 | 0.12 |
| RAVLT (Trial 6) | 0.03 | **-0.44** | 0.39 | -0.05 | 0.36 | 0.01 |
| SDMT (total score) | 0.18 | **-0.63** | 0.25 | 0.04 | 0.17 | **0.42** |
| PST (Dots) | -0.14 | **0.72** | -0.05 | 0.22 | -0.11 | -0.22 |
| PST (Interference) | -0.19 | **0.46** | -0.31 | 0.18 | -0.14 | **-0.46** |
| PST (Words) | -0.02 | **0.77** | -0.04 | 0.01 | -0.13 | 0.19 |
| TMT-A (total time) | -0.08 | **0.57** | -0.11 | 0.33 | 0.10 | -0.03 |
| TMT-B (total time) | -0.11 | **0.52** | -0.15 | 0.21 | -0.16 | **-0.43** |
| RAVLT (Total recall 1-5) | 0.24 | -0.15 | **0.81** | -0.12 | 0.22 | 0.11 |
| RAVLT (delayed recall) | 0.30 | -0.11 | **0.78** | -0.11 | 0.18 | 0.10 |
| RAVLT (Recognition) | 0.34 | 0.04 | **0.72** | -0.22 | 0.04 | 0.02 |
| LNS (total score) | 0.07 | -0.39 | **0.59** | 0.02 | 0.01 | 0.14 |
| GPT (left hand) | 0.01 | 0.10 | -0.11 | **0.83** | -0.04 | -0.20 |
| GPT (right hand) | 0.05 | 0.21 | -0.05 | **0.85** | -0.02 | -0.15 |
| MIST (time-based) | 0.20 | -0.18 | 0.36 | **-0.58** | 0.01 | -0.01 |
| VF (latter K) | 0.26 | -0.30 | -0.08 | -0.12 | **0.55** | -0.08 |
| VF (Action Verb) | 0.25 | -0.08 | 0.06 | 0.21 | **0.58** | 0.19 |
| VF (Vegetables) | 0.16 | 0.13 | 0.24 | -0.16 | **0.74** | 0.01 |
| VF (Animals/Clothes) | 0.17 | -0.38 | 0.17 | 0.01 | **0.61** | 0.19 |
| CDT (total score) | 0.00 | -0.17 | 0.08 | -0.13 | 0.26 | **0.66** |
| MoCA (cube) | 0.10 | 0.08 | 0.01 | -0.07 | -0.07 | **0.71** |
| MIST (event-based) | 0.18 | -0.23 | 0.21 | 0.25 | -0.07 | 0.24 |
| PST (Colours) | -0.29 | 0.25 | -0.13 | 0.22 | -0.08 | -0.36 |
|  |  |  |  |  |  |  |
| Proportion Explained | 0.28 | 0.18 | 0.17 | 0.13 | 0.13 | 0.11 |

*Note.* Factor loadings with a value greater than 0.40 are bolded. AM = Associative memory; PS/E = Processing speed/Executive functions; EM = Episodic memory; PS = Psychomotor speed; L= Language; VisF = Visuospatial functions; RAVLT = Rey Auditory Verbal Learning Test; MBT = Memory Binding Test; MIST = Memory for Intentions Screening Test; TMT = Trial Making Test; LNS = Letter-Number Sequencing from Wechsler Adult Intelligence Scale, Third Revision; PST = Prague Stroop Test; VF = Verbal fluency; CDT = Clock Drawing Test; GPT = Grooved Pegboard Test; MoCA = Montreal Cognitive Assessment; SDMT = Symbol Digit Modalities Test.

**Supplemental Table 4**
Detailed information about significant clusters in VBM correlation analysis

|  | Peak Localisation [mm mm mm] | T value (Peak) | Anatomical region (cluster, aal atlas) |
| --- | --- | --- | --- |
| iRBD vs. TMT-A cluster 1 | 35/-3/-8 | 4.68 | Right hemisphere: Putamen, Insula, Temporal Superior Lobe, Pallidum, Caudate, Amygdala |
| iRBD vs. TMT-A cluster 2 | -24/-50/-14 | 4.66 | Left hemisphere: Cerebellum, Fusiform, Lingual Gyrus |
| iRBD vs. TMT-B | -32/-5/-15 | 5.06 | Left hemisphere: Amygdala, Hippocampus, Parahippocampal Gyrus, Fusiform Gyrus |
| iRBD vs. GPT right hand | -32/-71/38 | 4.51 | Left Hemisphere: Occipital Middle and Superior Lobe, Parietal Superior and Inferior Lobe |
| iRBD vs. RAVLT 1-5 cluster 1 | -47/-24/42 | 4.68 | Left hemisphere: Postcentral Gyrus, Parietal Inferior Lobe, Precentral Gyrus, Supra Marginal Gyrus |
| iRBD vs. RAVLT 1-5 cluster 2 | 38/-6/-12 | 4.10 | Right hemispehre: Rolandic Operculum, Insula, Temporal Superior, Heschl, Hippocampus |
| HC vs. RAVLT 1-5 cluster 1 | 2/38/-6 | 4.38 | Left hemisphere: Cigulum Anterior, Olfactory Lobe, Frontal Middle Orb., Caudate  Right Hemisphere: Cingulum Anterior, Frontal Middle Orb |
| iRBD vs Psychomotor speed (PCA component) | -9/-59/-11 | 4.52 | Vermis  Left hemisphere: Cerebellum, Lingual Gyrus  Right Hemispere: Cerebellum |

*Note.* iRBD = Isolated rapid eye movement sleep behavior disorder; HC = healthy controls; TMT-A = Trail Making Test, part A; TMT-B = Trail Making Test, part B; GPT = Grooved Pegboard Test; RAVLT 1-5 = Rey Auditory Verbal Learning Test 1-5; PCA = principal components analysis.

**Supplemental Table 5**Detailed information about significant clusters in DBM correlation analysis

|  | Peak Localisation [mm mm mm] | T value (Peak) | Anatomical region (cluster, aal atlas) |
| --- | --- | --- | --- |
| iRBD vs. TMT-A | 38/-8/2 | 4.88 | Right hemisphere: Putamen, Pallidum, Insula, Caudate, Superior Temporal Lobe |
| iRBD vs. GPT right hand | -27/-83/21 | 4.98 | Left hemisphere: Middle and Superior Occipital Lobe, Superior Parietal Lobe |

*Note.* iRBD = Isolated rapid eye movement sleep behavior disorder; TMT-A = Trail Making Test, part A; GPT = Grooved Pegboard Test.

**Supplemental Table 6**
Correlation between performance in cognitive tests and the severity of REM sleep without atonia indexes in iRBD

|  |  | RWA index | | |
| --- | --- | --- | --- | --- |
|  | SINBAR score | Tonic | Phasic | Mixed |
| RAVLT (Total recall 1-5) | -0.062 | -0.063 | -0.021 | -0.147 |
| RAVLT (Trial 6) | -0.078 | -0.052 | 0.015 | -0.008 |
| RAVLT (delayed recall) | 0.037 | -0.004 | 0.144 | -0.043 |
| RAVLT (recognition) | -0.039 | -0.042 | 0.001 | -0.097 |
| MIST (event-based) | -0.086 | -0.130 | -0.016 | -0.170 |
| MIST (time-based) | -0.208 | -0.143 | -0.243 | -0.149 |
| MBT (Total Cued Recall) | -0.330 | -0.219 | -0.168 | -0.217 |
| MBT (Paired Recall Pairs) | -0.347 | -0.228 | -0.182 | -0.242 |
| MBT (Total Delayed Paired Recall) | -0.336 | -0.243 | -0.175 | -0.212 |
| MBT (Delayed Paired Recall Pairs) | -0.328 | -0.223 | -0.137 | -0.209 |
| MBT (Total Free Recall) | -0.287 | -0.284 | -0.089 | -0.305 |
| MBT (Total Delayed Free Recall) | -0.174 | -0.118 | -0.087 | -0.098 |
| LNS | -0.202 | -0.245 | -0.263 | -0.211 |
| TMT-A | -0.073 | 0.016 | -0.079 | -0.041 |
| PST (colors) | -0.175 | -0.054 | -0.099 | -0.173 |
| PST (interference) | -0.160 | -0.117 | -0.052 | -0.131 |
| TMT-B | -0.335 | -0.257 | -0.297 | -0.267 |
| VF (animals/clothes) | -0.188 | -0.178 | -0.097 | -0.071 |
| CDT | -0.068 | -0.010 | 0.069 | -0.057 |
| MoCA (cube) | 0.066 | 0.131 | 0.030 | 0.120 |
| GPT (left hand) | 0.062 | 0.048 | -0.085 | 0.105 |
| GPT (right hand) | -0.208 | -0.163 | -0.316 | -0.100 |
| SDMT | -0.096 | -0.096 | -0.107 | -0.047 |
| PST (Dots) | 0.017 | -0.021 | 0.107 | 0.011 |
| PST (words) | 0.030 | -0.047 | 0.085 | 0.100 |
| VF (K) | -0.093 | -0.043 | 0.105 | -0.071 |
| VF (action verb) | -0.108 | -0.125 | 0.023 | -0.092 |
| VF (vegetables) | -0.014 | 0.023 | 0.192 | -0.057 |

*Note.* N = 58. RWA = REM without atonia; RAVLT = Rey Auditory Verbal Learning Test; MBT = Memory Binding Test; MIST = Memory for Intentions Screening Test; TMT = Trial Making Test; LNS = Letter-Number Sequencing from Wechsler Adult Intelligence Scale, Third Revision; PST = Prague Stroop Test; VF = Verbal fluency; CDT = Clock Drawing Test; GPT = Grooved Pegboard Test; MoCA = Montreal Cognitive Assessment; SDMT = Symbol Digit Modalities Test.

Correlations with *p* values < 0.05 after Benjamini-Hochberg correction are in bold.
